# A Chest Patch for Continuous Vital Sign Monitoring: Clinical Validation Study During Movement and Controlled Hypoxia

**DOI:** 10.2196/27547

**Published:** 2021-09-15

**Authors:** Carlos Morgado Areia, Mauro Santos, Sarah Vollam, Marco Pimentel, Louise Young, Cristian Roman, Jody Ede, Philippa Piper, Elizabeth King, Owen Gustafson, Mirae Harford, Akshay Shah, Lionel Tarassenko, Peter Watkinson

**Affiliations:** 1 Critical Care Research Group, Nuffield Department of Clinical Neurosciences University of Oxford Oxford United Kingdom; 2 National Institute for Health Research, Biomedical Research Centre Oxford United Kingdom; 3 Institute of Biomedical Engineering, Department of Engineering Science University of Oxford Oxford United Kingdom; 4 Therapies Clinical Service Unit, Oxford University Hospitals NHS Foundation Trust Oxford United Kingdom; 5 Radcliffe Department of Medicine University of Oxford Oxford United Kingdom; 6 Kadoorie Centre for Critical Care Research and Education Oxford University Hospitals NHS Trust Oxford United Kingdom

**Keywords:** clinical validation, chest patch, vital signs, remote monitoring, wearable, heart rate, respiratory rate

## Abstract

**Background:**

The standard of care in general wards includes periodic manual measurements, with the data entered into track-and-trigger charts, either on paper or electronically. Wearable devices may support health care staff, improve patient safety, and promote early deterioration detection in the interval between periodic measurements. However, regulatory standards for ambulatory cardiac monitors estimating heart rate (HR) and respiratory rate (RR) do not specify performance criteria during patient movement or clinical conditions in which the patient’s oxygen saturation varies. Therefore, further validation is required before clinical implementation and deployment of any wearable system that provides continuous vital sign measurements.

**Objective:**

The objective of this study is to determine the agreement between a chest-worn patch (VitalPatch) and a gold standard reference device for HR and RR measurements during movement and gradual desaturation (modeling a hypoxic episode) in a controlled environment.

**Methods:**

After the VitalPatch and gold standard devices (Philips MX450) were applied, participants performed different movements in seven consecutive stages: at rest, sit-to-stand, tapping, rubbing, drinking, turning pages, and using a tablet. Hypoxia was then induced, and the participants’ oxygen saturation gradually reduced to 80% in a controlled environment. The primary outcome measure was accuracy, defined as the mean absolute error (MAE) of the VitalPatch estimates when compared with HR and RR gold standards (3-lead electrocardiography and capnography, respectively). We defined these as clinically acceptable if the rates were within 5 beats per minute for HR and 3 respirations per minute (rpm) for RR.

**Results:**

Complete data sets were acquired for 29 participants. In the movement phase, the HR estimates were within prespecified limits for all movements. For RR, estimates were also within the acceptable range, with the exception of the sit-to-stand and turning page movements, showing an MAE of 3.05 (95% CI 2.48-3.58) rpm and 3.45 (95% CI 2.71-4.11) rpm, respectively. For the hypoxia phase, both HR and RR estimates were within limits, with an overall MAE of 0.72 (95% CI 0.66-0.78) beats per minute and 1.89 (95% CI 1.75-2.03) rpm, respectively. There were no significant differences in the accuracy of HR and RR estimations between normoxia (≥90%), mild (89.9%-85%), and severe hypoxia (<85%).

**Conclusions:**

The VitalPatch was highly accurate throughout both the movement and hypoxia phases of the study, except for RR estimation during the two types of movements. This study demonstrated that VitalPatch can be safely tested in clinical environments to support earlier detection of cardiorespiratory deterioration.

**Trial Registration:**

ISRCTN Registry ISRCTN61535692; https://www.isrctn.com/ISRCTN61535692

## Introduction

### Background

Failure to recognize and act on physiological indicators of deterioration in hospital wards is a well-recognized problem [1-3]. Current practice involves manual measurement of vital signs and the use of early warning scores. However, the acquisition of these measurements can be time consuming for health professionals in a busy ward [4]; therefore, the desired monitoring frequency of observations is often not achieved [5]. This is identified as a limitation of periodic monitoring systems, as they are dependent on the frequency of vital sign observations [6]; of more concern is that even when the ideal frequency is achieved, patients might deteriorate between observation sets [7]. Continuous vital sign monitoring using wearable devices may overcome these limitations and improve timely detection of deterioration [8-10]. However, recent observational studies and pilot randomized controlled trials of wearable monitoring devices were inconclusive, and large randomized clinical trials of ambulatory monitoring devices are required to demonstrate improved patient outcomes [1,8,11,12]. This may be because of limitations in currently available ambulatory monitoring devices; for instance, it has been demonstrated that the detection and reduction of noise and artifacts during movement can be challenging in this type of monitoring [13]. Therefore, there is a need for diagnostic accuracy studies addressing motion artifacts to support the development of reliable wearable devices [3,9,14,15].

**Figure 1:**
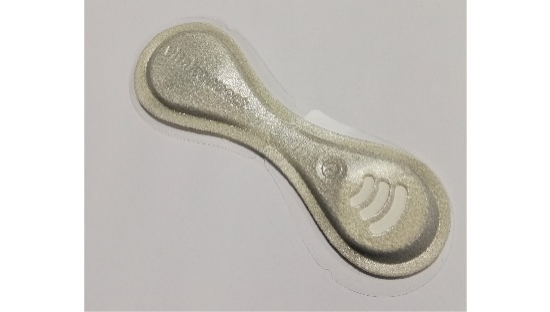
Picture of a VitalPatch device.

The VitalPatch [16] is a wireless, battery-operated chest-worn wearable biosensor that records heart rate (HR), electrocardiography (ECG), HR variability, R-R interval (the time elapsed between two successive R-waves of the QRS signal on the electrocardiogram), respiratory rate (RR), body temperature, skin temperature, fall detection, activity (including step count), and posture (body position relative to gravity including fall detection) [17,18]. This device (Figure 1) was well tolerated by users because of its wearability and low impact on activities [19,20]. However, validation studies regarding the VitalPatch showed mixed results [3]; for HR, the mean bias was within acceptable ranges for all validation studies [14,18,21-23] and within acceptable limits of agreement (LoA) in 3 studies [18,21,22]. For RR, the mean bias was also acceptable; however, all studies reported LoA outside of the clinically acceptable range [14,18,21-23].

This work is a substudy of our virtual high dependency unit (vHDU) hypoxia study [24]. In the main protocol, our goal was to determine the specificity and sensitivity of currently available ambulatory pulse oximeters for the detection of hypoxemia. In this substudy, the aim is to assess the VitalPatch estimation accuracy during several simulated movements and in controlled hypoxic conditions against gold standard measurements, ECG for HR [25] and capnography for RR [26].

### Objectives

The primary outcome is accuracy, defined as the mean absolute error (MAE) of the VitalPatch HR and RR estimation versus gold standard recording of HR (3-lead ECG) and RR (capnography) in healthy volunteers during movement and during induced hypoxia (down to peripheral oxygen saturation [SpO_2_] of 80%). In line with the American National Standards Institute/Association for the Advancement of Medical Instrumentation (ANSI/AAMI) EC13:2002 standard [27], we defined the MAE of the patch estimates to be clinically acceptable if the rates are within 5 beats per minute (bpm) and 3 respirations per minute (rpm) [3,21,22,27].

## Methods

This manuscript follows the Guidelines for Reporting Reliability and Agreement Studies [28].

### Study Design

This was a prospective, observational, cross-sectional cohort study that validated the HR and RR of a single-lead ECG chest patch. The VitalPatch HR is computed via the detection of Q wave, R wave, and S wave complexes from a single-lead ECG waveform. The RR is derived from combined information from three sources, that is, an embedded algorithm uses a weighted average of two characteristics of the ECG signal: (1) Q-, R-, and S-wave amplitude modulation and (2) respiratory sinus arrhythmia (as both ECG-derived signals change during inspiration and expiration), and (3) changes in the accelerometer signal induced by chest movement during respiration [23,29,30]. We compared the HR and RR estimates with the *gold standard* HR derived from 3-lead ECG and RR determined from capnography measurements, respectively. The protocol for the full study has been published [24].

### Participants

The screening process and inclusion and exclusion criteria are described in the study protocol [24]. Participants were recruited consecutively from the Oxford area, United Kingdom, between June 18 and August 8, 2019. The study sessions took place at the Cardiovascular Clinical Research Facility, John Radcliffe Hospital, Oxford, United Kingdom.

### Device Placement

After the participants provided written informed consent, an arterial line was inserted into the participant’s nondominant radial artery. The participants were then fitted with a VitalPatch in a modified lead-II configuration on the left midclavicular line over the intercostal space (as recommended by the vendor), a nonambulatory Philips MX 450 pulse oximeter (dominant arm), a 3-lead ECG (standard configuration) for acquisition of HR, and a nasal cannula for acquisition of RR, both connected to the Philips monitor. The participants were continuously monitored with these devices throughout the study session by trained health care professionals with appropriate resuscitation facilities.

### Test Methods

#### Stage 1: Movement Phase

The participants were considered *at rest* after fitting all the devices (variable duration between participants). The participants then moved into a chair and were asked to complete a series of consecutive motion conditions: 20× sit-to-stand (STS), 2-minute tapping at 2 Hz, 2-minute rubbing at 2 Hz, 20 times drinking from a plastic cup, 50 times turning pages, and a set of predefined table activity tasks [24]. STS was considered a dynamic activity, whereas the remainder were considered *sedentary activities*.

#### Stage 2: Hypoxia Exposure Phase

Following the movement phase, participants were placed in a semirecumbent, supine position and wore a tight-fitting silicone facemask connected to a hypoxicator unit (Everest Summit Hypoxic Generator). During this phase, oxygen saturations from the *clinical standard* Philips monitor guided the titration of the hypoxicator by a senior anesthetist from the research team. Oxygen (7%) in nitrogen was used to further lower the fraction of inspired oxygen, if required [24]. The inhaled fraction of inspired oxygen was also monitored using an in-line gas analyzer. Participants were gradually desaturated to reach stable prespecified target SpO_2_ levels of 95%, 90%, 87%, 85%, 83%, and 80%. These levels were selected to allow sufficient data points for different hypoxia levels. SpO_2_ stability was subjective for each target window, that is, a senior anesthetist decided when a stable oxygen level was achieved.

### Data Collection

For each participant, the following information was recorded in the case report form: age, sex, height, weight, skin type (Fitzpatrick scale [31]), baseline HR (Philips MX 450 3-lead ECG), and arterial oxygen saturation. Data from all participants were identified using a study number.

All data collection devices (wearables, tablets, laptops, and physiological monitors), and respective software, were synchronized to the same time server at the start of each study session day. Continuous HR and RR data (sampled at 0.25 Hz) from the VitalPatch were sent via Bluetooth Low Energy to an Android tablet–based data capture system, developed in-house. Once the VitalPatch is connected to the tablet, it updates its internal clock and timestamps each sample internally. The Phillips MX 450 3-lead ECG data were collected in real time on a laptop using the ixTrend version 2.1 software (ixcellence GmbH) [32]. For each step of the *movement phase* and prespecified oxygen saturation level of the *hypoxia phase*, the corresponding start and stop time stamps were recorded both in the in-house data capture software and on the respective case report forms [24].

### Data Analysis

#### Sample Size

Our sample size calculation was based on recommendations from the International Organization for Standardization 80601-2-61:2019 (as the primary objective of the original study was the assessment of pulse oximeter accuracy). For our secondary analysis of the VitalPatch, we aimed to have approximately 30 complete patient data sets to ensure sufficient data points for analysis and validation testing using the VitalPatch and gold standard (Philips monitor) continuous data.

#### Statistical Analysis

To analyze the agreement between the VitalPatch and the gold standard during the movement phase, we compared 40-second median HR and RR estimates from the VitalPatch with simultaneous 40-second median HR and RR estimates from the Phillips monitor from each movement task interval (±20 seconds at the start and at the end, respectively, to accommodate for the 40-second window size). For the hypoxia phase (100% to 80% saturations), 40-second median HR and RR estimates were also sampled for both devices from the start until the end of hypoxia testing. For each window, simultaneous 40-second median SpO_2_ estimates from the Philips monitor were also calculated per participant (our pulse oximetry hypoxia study suggested a negligible mean bias between the Philips SpO_2_ and arterial oxygen saturation). The 40-second window was chosen as the VitalPatch outputs instantaneous HR and RR estimates every 4 seconds, which gives 10 points per median estimate; the Philips monitor outputs both at 1 Hz. Therefore, this represents the comparison of a 10-point median with a 40-point median centered on the same sample, which is reasonable.

For each phase, descriptive summaries for both devices are shown (number of data points and mean and SD values of HR and RR) and the MAE and root mean square error (RMSE) were computed for these vital signs. However, MAE was the metric used to determine HR and RR accuracy, in accordance with the ANSI/AAMI EC13:2002 standard [27]. It was defined as clinically acceptable if ≤5 bpm and 3 rpm, respectively. Both MAE and RMSE 95% CIs were determined via bootstrap using 10,000 replicates [33].

Bland-Altman plots, the corresponding mean bias, and the 95% LoA (SD 1.96) were also analyzed [34]. Finally, the Pearson correlation coefficient and associated *P* value [35] were reported. All statistical tests were conducted using R v3.6.1 (R Foundation for Statistical Computing) [36].

#### Missing Values

Participants in whom it was not possible to achieve hypoxemia (SpO_2_<90%) were excluded from the analysis to avoid different sample sizes or participants in the movement and hypoxia phases. All the included participants had complete data from the movement phase. If there were any technical issues during an activity, it was repeated until complete data were obtained. For clinical reasons, this was not possible in the hypoxic phase. Participants with intermittent missing data (if not significant) were included. We did not use imputation in this study, given that the median was calculated for consecutive 40-second windows without data on either the VitalPatch or reference device excluded from the analysis.

## Results

### Participants

**Figure 2:**
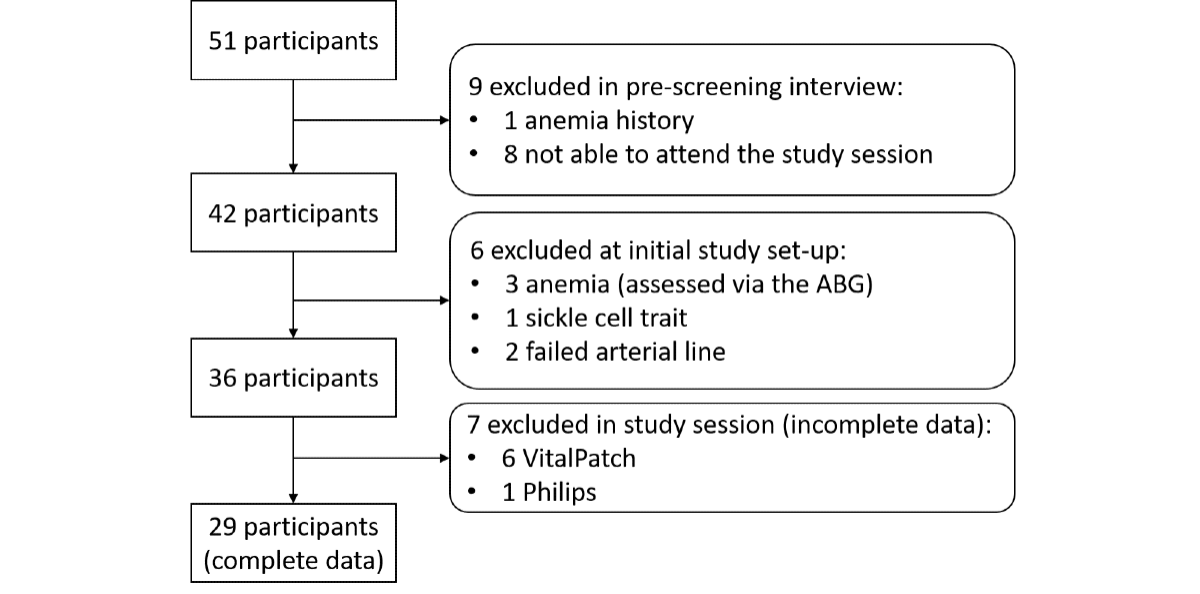
Participant flowchart. ABG: arterial blood gas.

The flow diagram of the data set collected in this study is shown in Figure 2. A total of 42 participants were recruited, of which 29 (69%) were included in our analysis. The demographic descriptors are listed in Table 1. Exemplar HR and RR traces collected in the movement and hypoxia phases for one participant are shown below.

**Table 1:** Participants’ demographics (N=29).

Characteristics			Values
**Age (years)**			
	Mean (SD)	32.5 (10.5)	
	Median (IQR)	29 (25-38)	
Sex (female), n (%)			16 (55)
**Height (m)**			
	Mean (SD)	1.7 (0.1)	
	Median (IQR)	1.7 (1.6-1.8)	
**Weight (kg)**			
	Mean (SD)	70.6 (14.1)	
	Median (IQR)	68 (59.2-83)	
**BMI (kg/m^2^)**			
	Mean (SD)	24.3 (3.9)	
	Median (IQR)	24 (21.5-26.8)	
**Skin tone, n (%)**			
	Type 1	7 (24)	
	Type 2	12 (41)	
	Type 3	2 (7)	
	Type 4	8 (28)	
**Respiratory rate (rpm^a^)**			
	Mean (SD)	16.3 (3.6)	
	Median (IQR)	17 (13-19)	
**Heart rate (bpm^b^)**			
	Value, mean (SD)	72.9 (11.9)	
	Value, median (IQR)	73 (64-82)	
**SaO_2_^c^**			
	Mean (SD)	99.3 (1.3)	
	Median (IQR)	100 (99-100)	
**Systolic BP^d^ (mm Hg)**			
	Mean (SD)	134.1 (20.0)	
	Median (IQR)	129 (123-146)	
**Diastolic BP (mm Hg)**			
	Mean (SD)	78.5 (11.2)	
	Median (IQR)	75 (70-87)	

^a^rpm: respirations per minute.

^b^bpm: beats per minute.

^c^SaO_2_: arterial oxygen saturation.

^d^BP: blood pressure.

### Movement Phase

**Figure 3:**
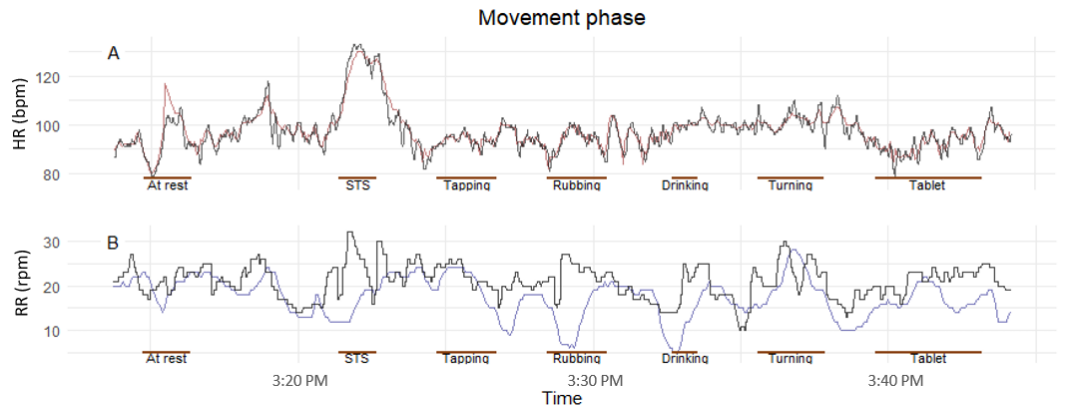
(A) Exemplar heart rate and (B) respiratory rate data collected during the movement phase for one participant (vHDU-H-029). Red: VitalPatch heart rate; blue: VitalPatch respiratory rate; black: Philips 3-Lead electrocardiography heart rate in part (A) and capnography respiratory rate in part (B); brown horizontal line: movement tests. bpm: beats per minute; HR: heart rate; rpm: respirations per minute; RR: respiratory rate; STS: sit-to-stand.

**Figure 4:**
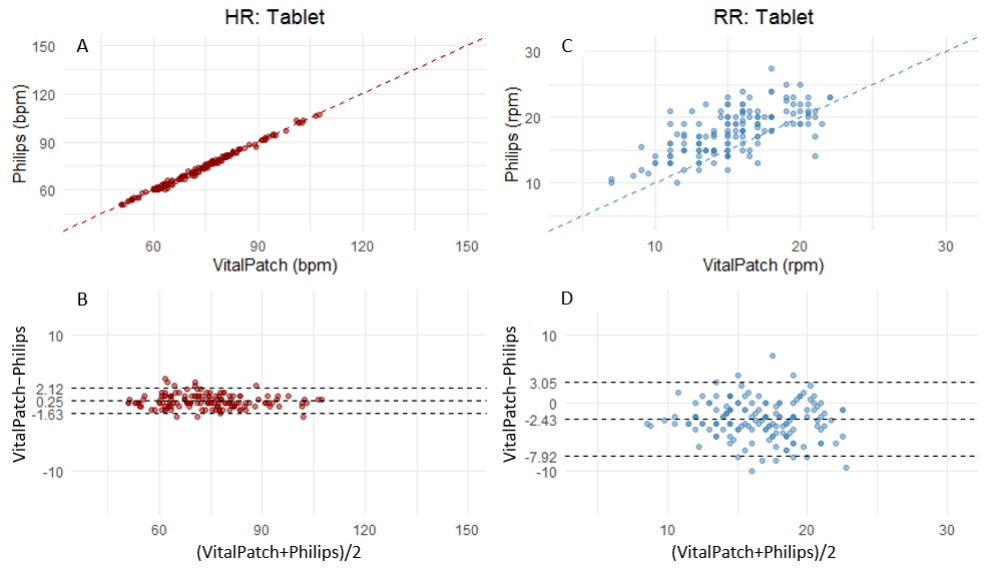
(A and C) Scatter plots and (B and D) Bland-Altman plots for heart rate (A and B in red) and respiratory rate (C and D in blue) in two example movements: (1) repetitive sit-to-stand and (2) using a tablet (while sitting). bpm: beats per minute; HR: heart rate; rpm: respirations per minute; RR: respiratory rate.

The movement phase lasted approximately 13 minutes for each participant. The exemplar HR and RR traces shown in Figure 3 confirm higher variability during the STS movement. The scatter and Bland-Altman plots for HR and RR in Figure 4 also show a higher mean bias and LoA for the dynamic activity (STS) versus one of the sitting movements (using a tablet). Scatter and Bland-Altman plots for all movement tests can be found in Multimedia Appendix 1. Table 2 lists the performance metrics for the complete movement phase. The VitalPatch HR showed marginal overestimation throughout the sitting activities, with a mean MAE ranging from 0.55-0.84 bpm, RMSE from 0.89-1.32 bpm, bias from 0.06-0.32 bpm, and with a correlation of approximately 1 (*P*<.001) throughout. In the dynamic activity (STS), there was an increase in the MAE, 2.69 (95% CI 1.58-3.63) bpm, and RMSE 5.75 (95% CI 3.83-7.94) bpm, mean bias 1.92 (95% LoA −8.75 to 12.59) bpm, with a correlation of approximately 0.95 (*P*<.001).

**Table 2:** Movement phase metrics (N=29).

Movement			Number of data points		Philips, mean (SD)		VitalPatch, mean (SD)		Pearson correlation (R^2^)^a^		MAE^b^ (95% CI)		RMSE^c^ (95% CI)		Mean bias (95% LoA^d^)
**Heart rate (bpm^e^)**															
	At rest	216		71.87 (12.52)		72.18 (12.86)		0.99		1.16 (0.91 to 1.39)		2.11 (1.62 to 2.62)		0.31 (−3.8 to 4.42)	
	STS^f^	93		89.94 (17.32)		91.87 (16.38)		0.95		2.69 (1.58 to 3.63)		5.75 (3.83 to 7.94)		1.92 (−8.75 to 12.59)	
	Tapping	111		70.99 (13.89)		71.07 (14.10)		1		0.55 (0.42 to 0.68)		0.89 (0.66 to 1.12)		0.08 (−1.68 to 1.83)	
	Rubbing	107		71.90 (12.69)		72.06 (12.69)		1		0.69 (0.51 to 0.85)		1.12 (0.88 to 1.37)		0.15 (−2.03 to 2.34)	
	Drinking	76		71.93 (13.91)		72.25 (13.68)		1		0.84 (0.61 to 1.05)		1.32 (0.98 to 1.67)		0.32 (−2.21 to 2.84)	
	Turning pages	98		75.48 (13.71)		75.54 (13.58)		1		0.72 (0.52 to 0.90)		1.21 (0.80 to 1.57)		0.06 (−2.32 to 2.44)	
	Tablet	152		73.18 (12.45)		73.43 (12.38)		1		0.69 (0.58 to 0.80)		0.99 (0.85 to 1.13)		0.25 (−1.63 to 2.12)	
**Respiratory rate (rpm^g^)**															
	At rest	127		17.26 (3.29)		15.31 (3.12)		0.72		2.44 (2.11 to 2.77)		3.10 (2.70 to 3.48)		−1.94 (−6.68 to 2.79)	
	STS	92		18.41 (4.71)		16.83 (5.20)		0.56		3.45 (2.71 to 4.11)		4.89 (3.91 to 5.92)		−1.59 (−10.7 to 7.53)	
	Tapping	104		18.43 (2.75)		16.75 (3.06)		0.68		2.14 (1.76 to 2.50)		2.86 (2.40 to 3.35)		−1.68 (−6.25 to 2.89)	
	Rubbing	103		17.50 (3.51)		15.52 (3.13)		0.63		2.44 (1.93 to 2.88)		3.48 (2.45 to 4.38)		−1.97 (−7.63 to 3.69)	
	Drinking	72		16.90 (3.76)		15.46 (3.48)		0.74		2.30 (1.83 to 2.72)		2.99 (2.36 to 3.62)		−1.44 (−6.62 to 3.75)	
	Turning pages	97		19.09 (4.08)		17.41 (3.72)		0.53		3.05 (2.48 to 3.58)		4.13 (3.32 to 4.90)		−1.69 (−9.11 to 5.73)	
	Tablet	152		17.75 (3.51)		15.32 (3.23)		0.66		2.97 (2.61 to 3.31)		3.70 (3.31 to 4.10)		−2.43 (−7.92 to 3.05)	

^a^*P*<.001.

^b^MAE: mean absolute error.

^c^RMSE: root mean square error.

^d^LoA: limits of agreement.

^e^bpm: beats per minute.

^f^STS: sit-to-stand.

^g^rpm: respirations per minute.

**Figure 5:**
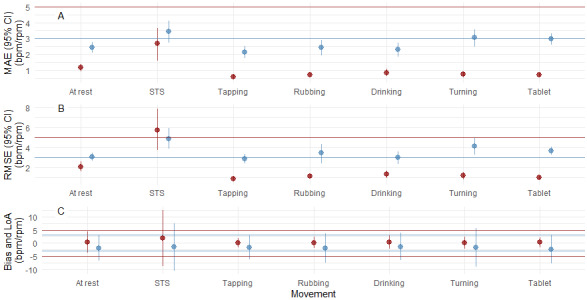
Accuracy and Bias (mean bias) plots for all movement tests. Red: heart rate; horizontal red line represents acceptable limits. Blue: respiratory rate; horizontal blue line represents acceptable limits. (A) MAE (95% CI) plot, (B) RMSE (95% CI) plot, (C) bias LOAs. bpm: beats per minute; LoAs: limits of agreement; MAE: mean absolute error; RMSE: root mean square error; rpm: respirations per minute; STS: sit-to-stand.

In contrast, a consistent underestimation was observed for RR throughout the sitting activities. MAE ranged from 2.14-3.05 rpm, RMSE from 2.86-4.13, mean bias from −1.68 to −2.43 rpm, and the correlation coefficient from 0.53-0.74 (*P*<.001). During the dynamic activity, the MAE was 3.45 (95% CI 2.71-4.11) rpm, RMSE was 4.89 (95% CI 3.91-5.92) rpm, mean bias was −1.59 (95% LoA −10.7 to 7.53) rpm, and the correlation was 0.56 (*P*<.001). An overview of the MAE, RMSE, and mean bias (LoA) for all movements is shown in Figure 5.

**Figure 6:**
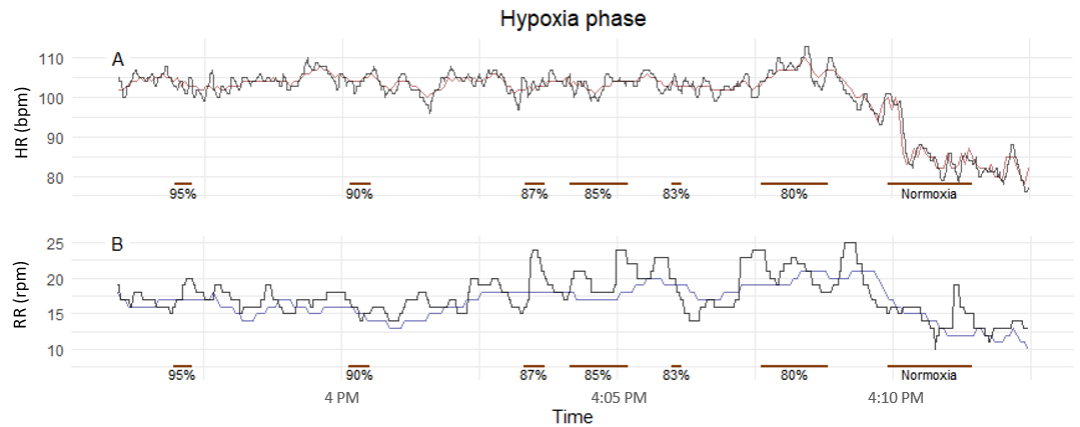
Exemplar heart rate (A) and respiratory rate (B) traces during the hypoxia phase for one participant (vHDU-H-29). red: VitalPatch heart rate; blue: VitalPatch respiratory rate; black: Philips 3-Lead electrocardiography heart rate (A) and capnography respiratory rate (B); brown horizontal line: target hypoxia levels. bpm: beats per minute; HR: heart rate; rpm: respirations per minute; RR: respiratory rate.

**Figure 7:**
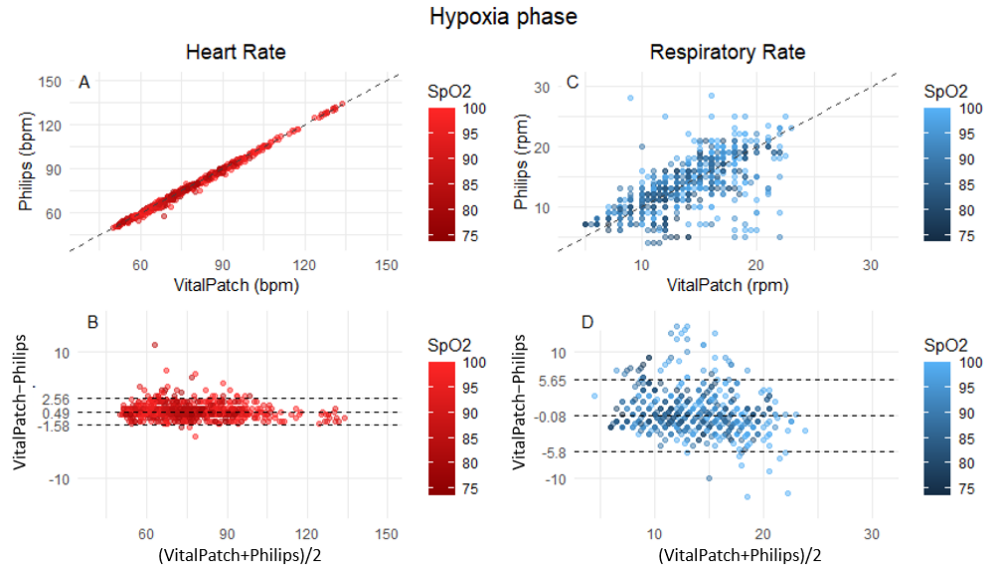
(A and C) Scatter plots and (B and D) Bland-Altman plots for heart rate (A and B in red) and respiratory rate (C and D in blue) during the full hypoxia phase. bpm: beats per minute; rpm: respirations per minute; SpO_2_: peripheral oxygen saturation.

The VitalPatch HR estimation was within clinically acceptable accuracy, achieving a MAE 0.72 (95% CI 0.66-0.78) bpm and a RMSE 1.16 (95% CI 1.02-1.29) bpm in the hypoxia phase. Figure 6 shows an exemplar participant during the hypoxia phase, and Figure 7 shows the scatter and Bland-Altman plots for HR and RR. The mean bias was 0.49 (95% LoA −1.58 to 2.56), and the correlation was approximately 1 (*P*<.001). Although more variable, the RR was also within the acceptable accuracy achieving a MAE 1.89 (95% CI 1.75-2.03) rpm and a RMSE 2.92 (95% CI 2.64-3.2) rpm. A minimal mean bias of −0.08 (95% LoA −5.8 to 5.65) and a correlation of approximately 0.72 (*P*<.001) were observed.

**Figure 8:**
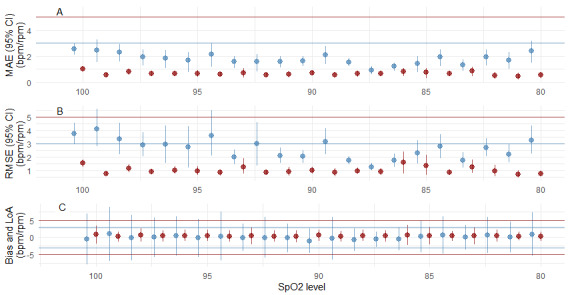
Accuracy and Bias (mean bias) plots for the hypoxia phase. Red: heart rate; horizontal red lines represent acceptable limits Blue: respiratory rate; horizontal blue line represents acceptable limits. (A) MAE (95% CI) plot, (B) RMSE (95% CI) plot, (C) bias LOAs. bpm: beats per minute; LoA:s limits of agreement; MAE: mean absolute error; RMSE: root mean square error; rpm: respirations per minute; SpO_2_: peripheral oxygen saturation.

There were also no significant differences in the error metrics between the SpO_2_ subgroups (normoxia, SpO_2_ 100% to 90%; mild hypoxia, SpO_2_ 85%-89.9%; and severe hypoxia, SpO_2_<85% [24]), as shown in Multimedia Appendix 2. Finally, Figure 8 shows the MAE, RMSE, and mean bias (LoA) computed for each oxygen saturation level from the hypoxia phase.

## Discussion

### Principal Findings

The key findings from this study were as follows: (1) The VitalPatch HR estimation was well within acceptable accuracy limits when compared with the gold standard device (Philips 3-lead ECG), (2) The VitalPatch RR estimation was also in agreement with the gold standard device (Philips capnography) for most of the movements and throughout the full hypoxia phase, and (3) there was no impact on the HR and RR estimation performance when participants were exposed to induced hypoxia. These findings support the clinical use of VitalPatch in patients with reduced baseline saturation or *at risk* of hypoxia.

Accuracy testing during motion is important before clinical use, as we aim to implement an ambulatory monitoring system that promotes patient mobility and independence without compromising safety. Our results suggest that the VitalPatch can measure HR with high accuracy and minimal bias during simple movements (mimicking in-hospital patient movements). Reliability was reduced during STS activity but was still within acceptable limits. These results agree with the results of previous clinical validation studies when compared with a reference standard device in healthy volunteers [18,23] and surgical patients [21,22]. When compared with nurse manual measurements in surgical patients [14], VitalPatch meets the accuracy criteria for HR estimation.

The MAE of the VitalPatch RR estimation was comparatively higher, especially during the movement phase. However, it was also mostly within the prespecified limits (except for the STS and turning a page movements), in agreement with previous studies [14,18,21-23]. High variation during STS was expected as it is a dynamic activity, potentially creating several artifacts that could affect VitalPatch accuracy during this test [21,22]; however, a high MAE for RR was not expected for the *turning pages* activity (even if close to the 3-rpm threshold). We postulate that the latter may have created a low-frequency periodic artifact that influenced both the ECG and accelerometer waveforms (both used in the computation of the RR estimate by the VitalPatch).

The most recent study showed that the VitalPatch tended to overestimate RR with a bias of 4.4 rpm [22], but previous studies showed a predominant RR underestimation [14,18,21]. Our study seems to agree with the latter, as VitalPatch mostly underestimated RR throughout the movement phase and varied in the hypoxia phase. However, in contrast to these previous studies, we collected vital signs from healthy participants.

This study also assessed the VitalPatch reliability during a controlled hypoxia phase, and our results suggest that there is no impact on this patch’s reliability for HR and RR measurements during periods of reduced SpO_2_ levels. The VitalPatch estimates followed the gold standard HR and RR variations caused by the controlled desaturation of the participants during the hypoxia phase, reliably, supporting its use in clinical populations with reduced baseline saturation or *at risk* of hypoxia.

### Limitations

This study had some limitations. First, the participants were healthy volunteers in a controlled environment that is not representative of hospitalized patients. In addition, the main study was powered to assess the accuracy of SpO_2_ and not that of HR and RR. Although the range required to validate RR was achieved (10 to 30 rpm is required [18] and 4 to 31 rpm was collected), the HR range achieved in this substudy was 40 to 150 bpm, a subset of the range required to validate its accuracy, 30 to 200 bpm [27].

### Comparison With Prior Work and Future Research

The ANSI/AAMI EC13:2002 standard [27] only requires that a measure of systematic error (eg, mean bias or MAE) be below the clinically accepted limits. In more recent studies, MAE has been used because it is less susceptible to outliers than the mean bias [18,23,30]. Analyzing the RMSE results, which combines both the systematic and random errors, we note that they follow the trend of those of the MAE metric as the RMSE was also higher during the STS activity for both variables and for the *turning pages* movement for RR. In our study, the LoA (also a measure of random error) seemed to be within the acceptable range when compared with the results of other studies [14,18,21-23] (except for the aforementioned cases). Previous studies reported higher LoA (defined as out of the acceptable range by the authors) for RR when compared with reference standard devices both in healthy volunteers (−10.1 to 6.5) [18] and surgical patients (−15.8 to 11.2) [21] and (−4.4 to 13.3) [22], and when compared with nurse manual measurements in surgical patients (10.32 to 9.04) [14].

In this study, the VitalPatch was subjected to robust testing, during movement and under induced hypoxia, against a gold standard in a controlled environment. Our results suggest that this device can be safely tested in clinical environments (within the collected input range for each vital sign). Our results will inform future phases of our vHDU research project, aiming to develop an accurate wearable system for monitoring ambulatory patients in hospitals.

### Conclusions

In conclusion, the VitalPatch was highly accurate throughout movement tests except for the measurement of RR during the STS activity and the *turning pages* movement. This device was also reliable throughout the hypoxia phase, with no significant MAE or bias differences in hypoxic levels. This study demonstrated that VitalPatch can be safely tested in clinical environments to support earlier detection of clinical deterioration.

## Appendix

Multimedia Appendix 1 Scatter and Bland-Altman plots for movement phase.

Multimedia Appendix 2 Hypoxia phase subgroups analysis.

## Abbreviations

ANSI/AAMI: American National Standards Institute/Association for the Advancement of Medical Instrumentation
bpm: beats per minute
ECG: electrocardiography
HR: heart rate
LoA: limits of agreement
MAE: mean absolute error
NIHR: National Institute for Health Research
RMSE: root mean square error
rpm: respirations per minute
RR: respiratory rate
SpO_2_: peripheral oxygen saturation
STS: sit-to-stand
vHDU: virtual high dependency unit
