# Supplementary material for: A Chest Patch for Continuous Vital Sign Monitoring: Clinical Validation Study During Movement and Controlled Hypoxia
Source: J Med Internet Res. 2021 Sep 15;23(9):e27547. doi: 10.2196/27547 (PMC8482195; doi:10.2196/27547)
Supplement: Multimedia Appendix 1 [file jmir_v23i9e27547_app1.docx]

# Multimedia appendix 1 – Scatter and Bland Altman Plots for movement phase

### At rest


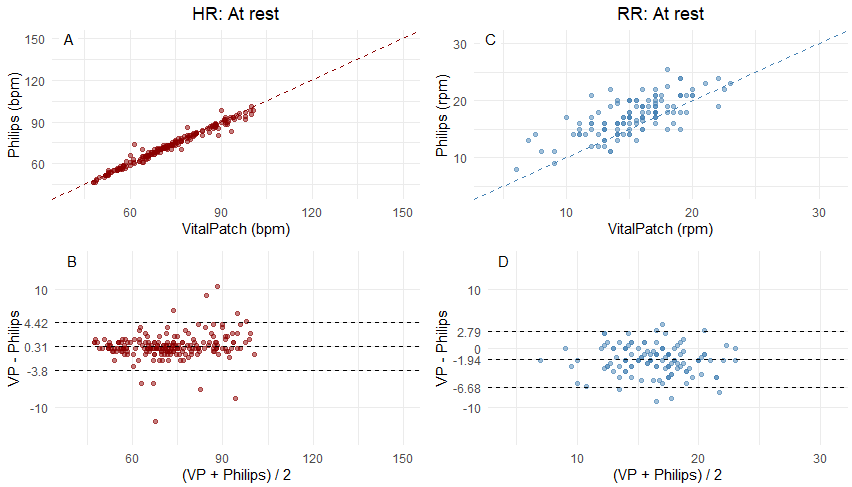


### Tapping


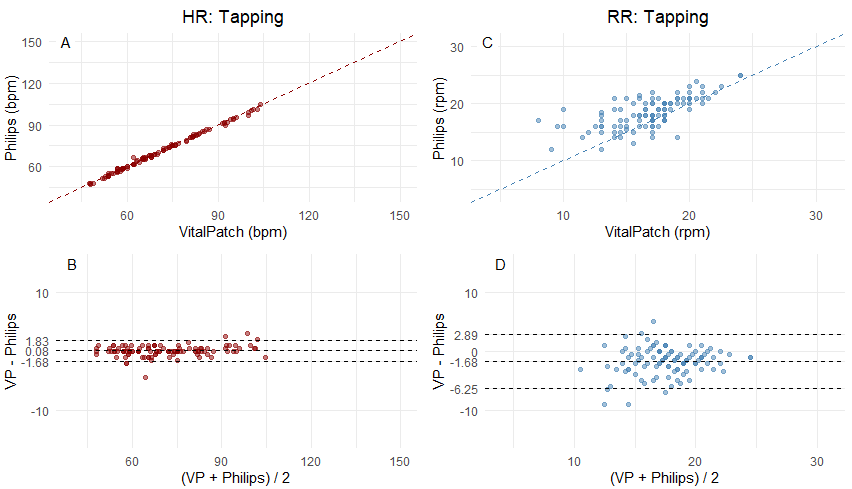


### Rubbing movement


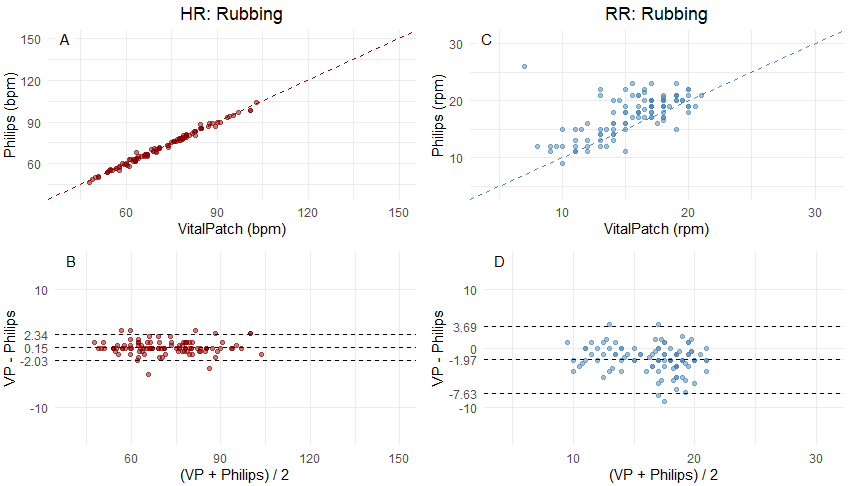


### Drinking movement


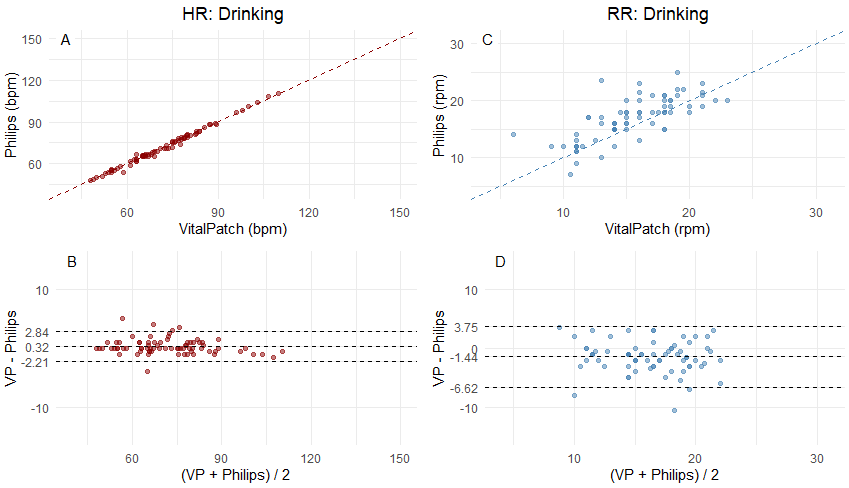


### Turning page movement


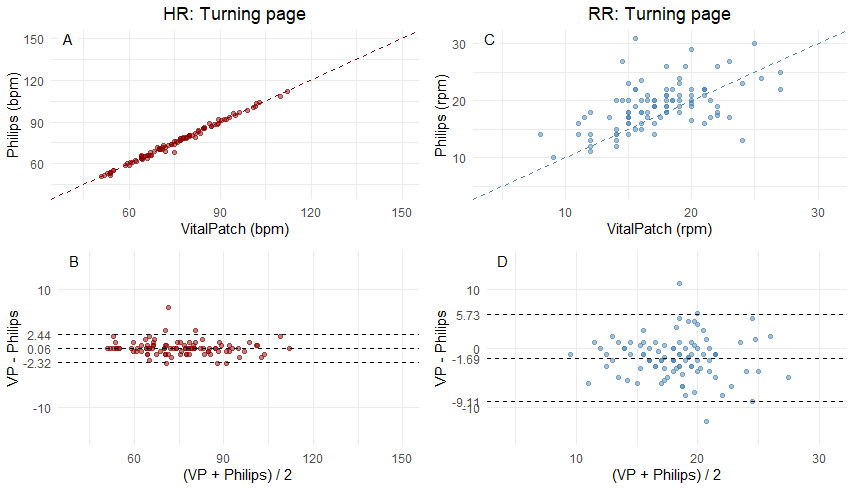


Figure 9 – Scatter plots (A and C) and Bland Altman plots (B and D) for heart rate (A and B in red) and respiratory rate (C and D in blue) for remaining movements. bpm: beats per minute, HR: Heart Rate, rpm: respirations per minute, RR: Respiratory rate, VP: VitalPatch
