# Supplementary material for: A Chest Patch for Continuous Vital Sign Monitoring: Clinical Validation Study During Movement and Controlled Hypoxia
Source: J Med Internet Res. 2021 Sep 15;23(9):e27547. doi: 10.2196/27547 (PMC8482195; doi:10.2196/27547)
Supplement: Multimedia Appendix 2 [file jmir_v23i9e27547_app2.docx]

# Multimedia appendix 2 – Hypoxia phase sub-groups analysis


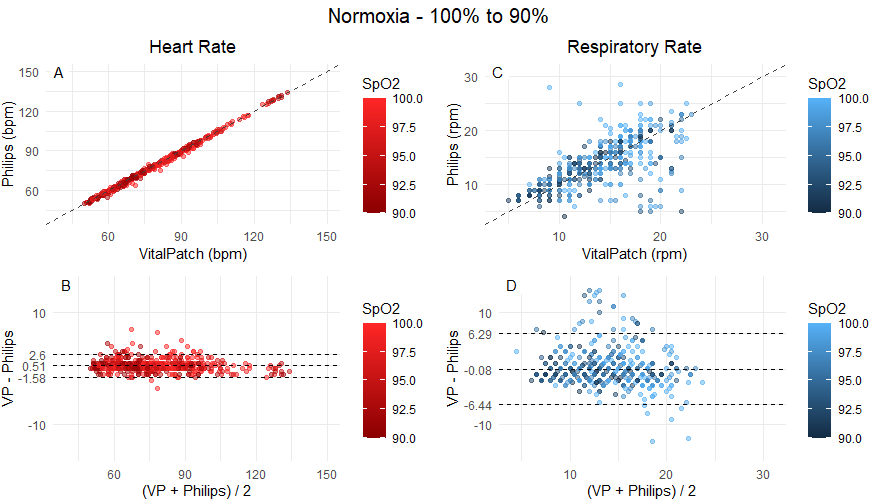


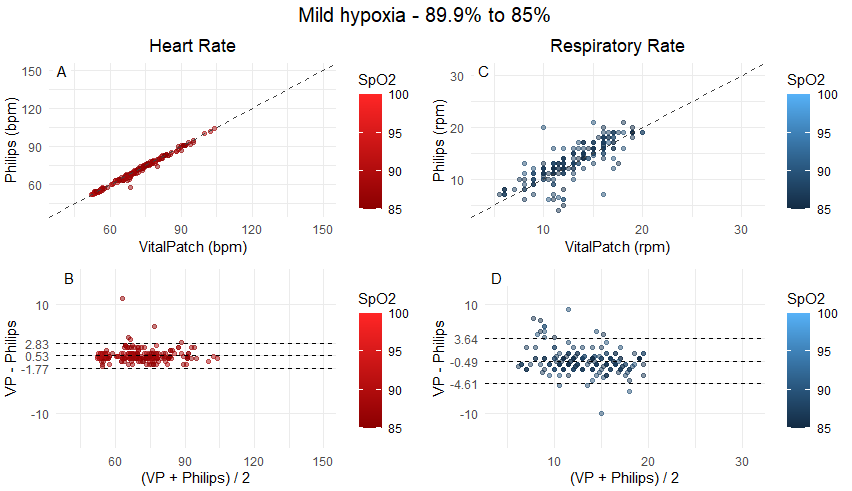

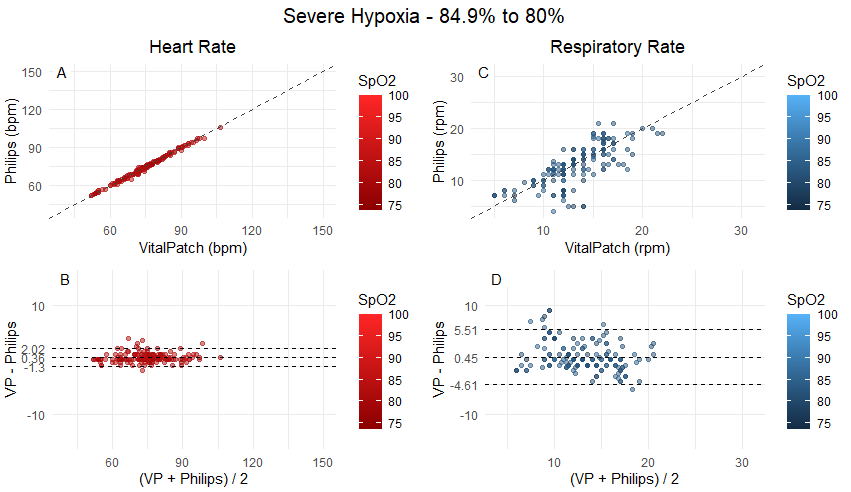


Figure 10 -Scatter plots (A and C) and Bland Altman plots (B and D) for heart rate (A and B in red) and respiratory rate (C and D in blue) grouped by normoxia (SpO2 = [90-100%]), %), mild-hypoxia (SpO2 = [85-89.9%]) and severe-hypoxia (SpO2 < 85). bpm: beats per minute, rpm: respirations per minute, SpO2 – Peripheral oxygen saturation, VP: VitalPatch

Table 3 and 4 show the statistics for HR and RR, respectively, during the full Hypoxia phase, the three SpO2 subgroups and individual rounded SpO2 levels.

| Heart rate (bpm^a^) | | | | | | | |
| --- | --- | --- | --- | --- | --- | --- | --- |
| SpO2^b^ (%) | **n^c^** | **Philips Mean±SD** | **VP^d^**  **Mean ±SD** | **Pearson Correlation** | **MAE^e^ (95% CI)** | **RMSE^f^ (95% CI)** | **Mean bias (95% LoA^g^)** |
| 100-80 | **989** | **74.43 ± 14.76** | **74.91±14.7** | **R^2h^=1*** | **0.72 (0.66, 0.78)** | **1.16 (1.02, 1.29)** | **0.49 (-1.58, 2.56)** |
| 100-90 | 590 | 75.79±16.85 | 76.3±16.76 | R^2^=1* | 0.76 (0.69, 0.83) | 1.18 (1.05, 1.30) | 0.51 (-1.58, 2.60) |
| 100 | 173 | 79.78±14.01 | 80.53±13.89 | R^2^=1* | 1.03 (0.85, 1.20) | 1.56 (1.27, 1.84) | 0.76 (-1.92, 3.43) |
| 99 | 56 | 76.72±18.35 | 76.91±18.29 | R^2^=1* | 0.56 (0.41, 0.71) | 0.79 (0.65, 0.95) | 0.19 (-1.34, 1.71) |
| 98 | 50 | 79.58±17.35 | 80.17±17.15 | R^2^=1* | 0.79 (0.54, 1.01) | 1.16 (0.83, 1.47) | 0.59 (-1.38, 2.56) |
| 97 | 46 | 78.57±19.47 | 78.96±19.47 | R^2^=1* | 0.65 (0.46, 0.84) | 0.93 (0.73, 1.15) | 0.39 (-1.27, 2.06) |
| 96 | 46 | 75.03±18.19 | 75.59±18.23 | R^2^=1* | 0.68 (0.46, 0.89) | 1.03 (0.77, 1.31) | 0.55 (-1.16, 2.27) |
| 95 | 29 | 77.64±22.01 | 78.19±21.71 | R^2^=1* | 0.66 (0.38, 0.90) | 0.98 (0.66, 1.32) | 0.55 (-1.07, 2.17) |
| 94 | 38 | 73.78±20.50 | 74.13±20.26 | R^2^=1* | 0.62 (0.42, 0.80) | 0.86 (0.65, 1.08) | 0.36 (-1.21, 1.92) |
| 93 | 33 | 69.56±15.74 | 69.95±15.44 | R^2^=1* | 0.73 (0.33, 1.06) | 1.29 (0.75, 1.91) | 0.39 (-2.04, 2.83) |
| 92 | 53 | 69.82±13.57 | 70.15±13.41 | R^2^=1* | 0.58 (0.39, 0.75) | 0.87 (0.68, 1.07) | 0.33 (-1.27, 1.93) |
| 91 | 41 | 67.52±13.32 | 67.82±13.15 | R^2^=1* | 0.59 (0.35, 0.79) | 0.92 (0.62, 1.24) | 0.29 (-1.45, 2.03) |
| 90 | 42 | 68.76±11.51 | 69.35±11.45 | R^2^=1* | 0.73 (0.51, 0.93) | 1.01 (0.79, 1.24) | 0.58 (-1.05, 2.22) |
| 89.9-85 | 224 | 70.97±10.66 | 71.5±10.64 | R^2^=0.99* | 0.73 (0.58, 0.85) | 1.28 (0.85, 1.65) | 0.53 (-1.77, 2.83) |
| 89 | 43 | 69.14±13.03 | 69.45±12.87 | R^2^=1* | 0.57 (0.36, 0.74) | 0.87 (0.54, 1.15) | 0.31 (-1.29, 1.91) |
| 88 | 38 | 74.26±11.03 | 74.82±11.11 | R^2^=1* | 0.66 (0.41, 0.88) | 0.99 (0.75, 1.25) | 0.55 (-1.07, 2.18) |
| 87 | 36 | 68.64±8.57 | 69.01±8.6 | R^2^=0.99* | 0.68 (0.46, 0.89) | 0.93 (0.69, 1.19) | 0.38 (-1.32, 2.07) |
| 86 | 74 | 72.86±9.82 | 73.51±9.72 | R^2^=0.99* | 0.80 (0.44, 1.07) | 1.61 (0.75, 2.39) | 0.64 (-2.28, 3.56) |
| 85 | 36 | 70.40±10.13 | 70.94±10.32 | R^2^=0.99* | 0.74 (0.29, 1.07) | 1.38 (0.69, 2.15) | 0.54 (-1.99, 3.07) |
| 84.9-80 | 175 | 74.25±10.4 | 74.61±10.49 | R^2^=1* | 0.58 (0.47, 0.69) | 0.92 (0.76, 1.07) | 0.36 (-1.30, 2.02) |
| 84 | 40 | 73.4±10.1 | 73.78±10.26 | R^2^=1* | 0.65 (0.48, 0.81) | 0.86 (0.67, 1.07) | 0.38 (-1.16, 1.91) |
| 83 | 28 | 73.54±11.1 | 73.91±11.2 | R^2^=0.99* | 0.84 (0.46, 1.18) | 1.29 (0.83, 1.81) | 0.38 (-2.09, 2.84) |
| 82 | 38 | 75.78±9.92 | 76.18±9.87 | R^2^=1* | 0.51 (0.24, 0.75) | 0.96 (0.58, 1.39) | 0.41 (-1.31, 2.13) |
| 81 | 21 | 78.05±11.6 | 78.4±11.71 | R^2^=1* | 0.45 (0.19, 0.69) | 0.73 (0.46, 1.03) | 0.36 (-0.93, 1.64) |
| 80 | 28 | 73.25±8.8 | 73.54±8.79 | R^2^=1* | 0.54 (0.32, 0.73) | 0.77 (0.56, 0.98) | 0.29 (-1.14, 1.71) |

Table 3- Hypoxia phase metrics table for Heart Rate.

^a^ bpm: beats per minute

^b^ SpO2: Peripheral oxygen saturation

^c^ n: number of datapoints

^d^ VP: VitalPatch

^e^ MAE: Mean Absolute Error

^f^ RMSE: Root Mean Squared Error

^g^ LoA: Limits of Agreement

^h^ R^2^: Coefficient of determination

* P < .001

| Respiratory Rate (rpm^a^) | | | | | | | |
| --- | --- | --- | --- | --- | --- | --- | --- |
| SpO2^b^ (%) | **n^c^** | **Philips Mean±SD** | **VP^d^**  **Mean ±SD** | **Pearson Correlation** | **MAE^e^ (95% CI)** | **RMSE^f^ (95% CI)** | **Mean bias (95% LoA^g^)** |
| 100-80 | **958** | **13.52±4.04** | **13.44±3.62** | **R^2^=0.72*** | **1.89 (1.75, 2.03)** | **2.92 (2.64, 3.20)** | **-0.08 (-5.80, 5.65)** |
| 100-90 | 566 | 13.72±4.27 | 13.65±3.91 | R^2^=0.69* | 2.07 (1.86, 2.27) | 3.25 (2.83, 3.65) | -0.08 (-6.44, 6.29) |
| 100 | 155 | 14.82±4.97 | 14.35±3.95 | R^2^=0.67* | 2.55 (2.09, 2.97) | 3.76 (2.96, 4.54) | -0.47 (-7.82, 6.87) |
| 99 | 53 | 14.38±4.75 | 15.38±4.19 | R^2^=0.60* | 2.47 (1.55, 3.27) | 4.11 (2.78, 5.57) | 1.00 (-6.88, 8.88) |
| 98 | 50 | 14.06±3.82 | 13.91±3.64 | R^2^=0.59* | 2.29 (1.58, 2.91) | 3.34 (2.26, 4.55) | -0.15 (-6.76, 6.46) |
| 97 | 45 | 13.31±3.23 | 13.31±3.64 | R^2^=0.64* | 1.96 (1.30, 2.53) | 2.89 (2.00, 3.85) | 0.00 (-5.72, 5.72) |
| 96 | 44 | 13.55±3.87 | 13.92±3.42 | R^2^=0.68* | 1.85 (1.08, 2.43) | 2.95 (1.51, 4.31) | 0.38 (-5.42, 6.17) |
| 95 | 29 | 13.55±3.88 | 13.38±4.09 | R^2^=0.75* | 1.66 (0.74, 2.29) | 2.76 (1.24, 4.27) | -0.17 (-5.67, 5.32) |
| 94 | 38 | 12.47±3.99 | 12.82±4.08 | R^2^=0.60* | 2.16 (1.16, 2.97) | 3.60 (2.10, 5.49) | 0.34 (-6.77, 7.46) |
| 93 | 33 | 12.65±3.6 | 12.58±3.68 | R^2^=0.84* | 1.56 (1.09, 1.97) | 2.02 (1.52, 2.56) | -0.08 (-4.09, 3.94) |
| 92 | 53 | 12.04±3.55 | 11.95±3.88 | R^2^=0.67* | 1.59 (0.81, 2.15) | 3.00 (1.39, 4.59) | -0.08 (-6.02, 5.85) |
| 91 | 41 | 12.85±3.96 | 12.63±3.66 | R^2^=0.85* | 1.56 (1.11, 1.98) | 2.11 (1.54, 2.68) | -0.22 (-4.39, 3.95) |
| 90 | 42 | 14.27±3.36 | 13.27±3.14 | R^2^=0.85* | 1.62 (1.23, 1.98) | 2.04 (1.54, 2.52) | -1.00 (-4.52, 2.52) |
| 89.9-85 | 221 | 13.5±3.56 | 13.01±3.12 | R^2^=0.81* | 1.47 (1.25, 1.67) | 2.16 (1.81, 2.50) | -0.49 (-4.61, 3.64) |
| 89 | 43 | 13.26±3.78 | 12.98±3.18 | R^2^=0.60* | 2.12 (1.36, 2.76) | 3.15 (2.22, 4.18) | -0.28 (-6.50, 5.94) |
| 88 | 38 | 14.17±3.46 | 13.55±2.97 | R^2^=0.87* | 1.51 (1.22, 1.82) | 1.77 (1.47, 2.07) | -0.62 (-3.92, 2.68) |
| 87 | 34 | 12.79±3.44 | 12.22±3.41 | R^2^=0.94* | 0.93 (0.63, 1.21) | 1.27 (1.04, 1.55) | -0.57 (-2.84, 1.69) |
| 86 | 74 | 13.51±3.45 | 12.96±3.08 | R^2^=0.87* | 1.21 (0.89, 1.49) | 1.78 (1.29, 2.25) | -0.55 (-3.90, 2.80) |
| 85 | 35 | 13.1±4.03 | 13.34±2.69 | R^2^=0.83* | 1.41 (0.77, 1.99) | 2.33 (1.54, 3.25) | 0.24 (-4.36, 4.84) |
| 84.9-80 | 171 | 12.87±3.82 | 13.32±3.16 | R^2^=0.74* | 1.83 (1.54, 2.11) | 2.61 (2.24, 2.98) | 0.45 (-4.61, 5.51) |
| 84 | 38 | 12.72±3.57 | 13.36±2.3 | R^2^=0.64* | 1.92 (1.24, 2.53) | 2.78 (1.93, 3.66) | 0.63 (-4.75, 6.02) |
| 83 | 28 | 12.91±3.82 | 12.93±3.86 | R^2^=0.89* | 1.34 (0.87, 1.75) | 1.78 (1.24, 2.37) | 0.02 (-3.54, 3.57) |
| 82 | 38 | 12.87±3.77 | 13.5±3.02 | R^2^=0.71* | 1.92 (1.29, 2.50) | 2.71 (2.04, 3.43) | 0.63 (-4.61, 5.87) |
| 81 | 20 | 15.12±3.46 | 15.25±3.61 | R^2^=0.79* | 1.68 (1.00, 2.30) | 2.23 (1.61, 2.95) | 0.12 (-4.36, 4.61) |
| 80 | 27 | 11.37±3.91 | 12.24±3.34 | R^2^=0.62* | 2.39 (1.48, 3.19) | 3.27 (2.25, 4.37) | 0.87 (-5.43, 7.17) |

Table 4 - Hypoxia phase metrics table for Respiratory Rate.

^a^ bpm: beats per minute

^b^ SpO2: Peripheral oxygen saturation

^c^ n: number of datapoints

^d^ VP: VitalPatch

^e^ MAE: Mean Absolute Error

^f^ RMSE: Root Mean Squared Error

^g^ LoA: Limits of Agreement

^h^ R^2^: Coefficient of determination

* P < .001
